# Preparation and Optimization of Garlic Oil/Apple Cider Vinegar Nanoemulsion Loaded with Minoxidil to Treat Alopecia

**DOI:** 10.3390/pharmaceutics13122150

**Published:** 2021-12-14

**Authors:** Waleed Y. Rizg, Khaled M. Hosny, Samar S. Elgebaly, Abdulmohsin J. Alamoudi, Raed I. Felimban, Hossam H. Tayeb, Majed Alharbi, Haitham A. Bukhary, Walaa A. Abualsunun, Alshaimaa M. Almehmady, Rasha A. Khallaf

**Affiliations:** 1 Department of Pharmaceutics, Faculty of Pharmacy, King Abdulaziz University, Jeddah 21589, Saudi Arabia; kmhomar@kau.edu.sa (K.M.H.); wabuassonon@kau.edu.sa (W.A.A.); amnalmehmady@kau.edu.sa (A.M.A.); 2 Center of Excellence for Drug Research and Pharmaceutical Industries, King Abdulaziz University, Jeddah 21589, Saudi Arabia; 3 Department of Clinical Biochemistry, Jouf University, Sakaka 71234, Saudi Arabia; samar_elgebaly@yahoo.com; 4 Department of Pharmacology and Toxicology, Faculty of Pharmacy, King Abdulaziz University, Jeddah 21589, Saudi Arabia; ajmalamoudi@kau.edu.sa; 5 Department of Medical Laboratory Technology, Faculty of Applied Medical Sciences, King Abdulaziz University, Jeddah 21589, Saudi Arabia; faraed@kau.edu.sa (R.I.F.); hhtayeb@kau.edu.sa (H.H.T.); 6 Center of Innovation in Personalized Medicine (CIPM), 3D Bioprinting Unit, King Abdulaziz University, Jeddah 21589, Saudi Arabia; 7 Center of Innovation in Personalized Medicine (CIPM), Nanomedicine Unit, King Abdulaziz University, Jeddah 21589, Saudi Arabia; 8 Department of Pharmaceutical Chemistry, Faculty of Pharmacy, King Abdulaziz University, Jeddah 21589, Saudi Arabia; maaalharbi1@kau.edu.sa; 9 Department of Pharmaceutics, Collage of Pharmacy, Umm Al-Qura University, Makkah 24381, Saudi Arabia; habukhary@uqu.edu.sa; 10 Department of Pharmaceutics and Industrial Pharmacy, Faculty of Pharmacy, Beni-Suef University, Beni-Suef 62511, Egypt; rasha.mahmoud@pharm.bsu.edu.eg

**Keywords:** minoxidil, garlic oil, nanoemulsion, optimization, alopecia areata, ex vivo permeation

## Abstract

Alopecia areata is a scarless, localized hair loss disorder that is typically treated with topical formulations that ultimately only further irritate the condition. Hence, the goal of this study was to develop a nanoemulsion with a base of garlic oil (GO) and apple cider vinegar (APCV) and loaded with minoxidil (MX) in order to enhance drug solubilization and permeation through skin. A distance coordinate exchange quadratic mixture design was used to optimize the proposed nanoemulsion. Span 20 and Tween 20 mixtures were used as the surfactant, and Transcutol was used as the co-surfactant. The developed formulations were characterized for their droplet size, minoxidil steady-state flux (MX Jss) and minimum inhibitory concentration (MIC) against *Propionibacterium acnes*. The optimized MX-GO-APCV nanoemulsion had a droplet size of 110 nm, MX Jss of 3 μg/cm^2^ h, and MIC of 0.275 μg/mL. The optimized formulation acquired the highest ex vivo skin permeation parameters compared to MX aqueous dispersion, and varying formulations lacked one or more components of the proposed nanoemulsion. GO and APCV in the optimized formulation had a synergistic, enhancing activity on the MX permeation across the skin membrane, and the percent permeated increased from 12.7% to 41.6%. Finally, the MX-GO-APCV nanoemulsion followed the Korsmeyer–Peppas model of diffusion, and the value of the release exponent (n) obtained for the formulations was found to be 1.0124, implying that the MX permeation followed Super case II transport. These results demonstrate that the MX-GO-APCV nanoemulsion formulation could be useful in promoting MX activity in treating alopecia areata.

## 1. Introduction

Alopecia areata is an ailment identified by impermanent, nonscarring hair loss in which the hair follicles are preserved [1]. In such a disorder, hair loss varies from well-defined spots to prevalent or total hair loss, which could influence all hairy parts of the body [2]. The most common type of alopecia areata is the inchoate alopecia areata that affects the scalp. About 2% of the general population suffers from alopecia areata at some point in the course of their lives, which often affects their self-esteem. resulting in disaffection and even depression [3]. Historically speaking, variable assumptions were assigned as triggers for alopecia areata. Immune disorders were considered the major cause of the disease during the past fifty years [4]. However, other reasons for alopecia areata, such as physical or emotional trauma, intoxication with thallium acetate, thyroid gland disorders, and microbial infections were also identified [5].

*Propionibacterium acnes* is a Gram-positive anaerobic commensal that lives on human skin and is implicated in acne and alopecia areata pathogenesis [6]. Curiously, when *P. acnes* feeds upon sebaceous glands, it produces porphyrins. These porphyrins are subsequently activated by UV radiation and release reactive oxygen species (ROS) [7]. ROSs are unstable oxygen-containing molecules, so they instantly engage with other nearby molecules. Such action of ROSs could lead to some unfortunate events, such as enzymatic destabilization and/or cellular degradation ending up with inflammation [8]. Intriguingly, porphyrins released in the sebaceous canals play a key role in the hair loss process by inducing chronic inflammation, which could participate in the chain of events leading to hair loss [9].

Minoxidil (MX) was originally developed as an antihypertensive drug; however, coincidently, practitioners observed an abnormal hair growth and generalized hirsutism during treatment, especially in bald patients [10]. Such a discovery led to the use of MX in topical treatment of different types of alopecia [11]. The proposed mechanism of action for this kind of treatment is thought to be through its vasodilating effect, which promotes blood flow to hair follicles and results in dermal papilla cell proliferation [12]. Nevertheless, MX also produces several negative effects (e.g., redness, inflammation, and itching) that restrict its long-term use, especially when it requires a dose twice a day [13]. Moreover, because MX has poor solubility and limited skin permeability, most of its commercial formulations are dissolved using varying proportions of different organic solvents (e.g., ethanol and propylene glycol (PG)) that dissolve MX at levels that result in sufficient hair growth [14]. Unfortunately, these organic solvents can impart unwanted effects, such as contact dermatitis, burning sensations, or scalp dryness [15]. Such drawbacks of marketed formulations containing MX necessitates scrutinizing new drug delivery systems and incorporating MX with other agents in order to administer MX with minimal side effects.

Garlic (Allium sativum) is an ancient plant, originally cultivated and used in Asia more than 600 years ago [16]. Garlic essential oil (GO) is rich in sulfuric compounds that have been reported to have several beneficial medicinal effects, including hypotensive, antitumor, antimicrobial, immunomodulatory, and even hair growth effects [17,18]. The anti-microbial properties of GO help kill fungi and bacteria that could damage the scalp and suppress hair growth. Additionally, raw garlic is rich in vitamin C, which enhances hair-growth and promotes collagen production [19]. Furthermore, GO’s selenium content improves blood circulation to hair follicles for ultimate nourishment [20].

Apple cider vinegar (APCV) is an amber-colored vinegar obtained from apples [21]. Chemically, APCV is described as a diluted aqueous solution of ethanoic acid that is created through alcoholic and acidic fermentation using acetobacter [22]. APCV works as a natural antifungal agent that may inhibit the growth of the dandruff-causing fungus, Malassezia furfur, therefore decreasing or diminishing dandruff [23]. APCV is considered an economic hair shampoo and conditioner, because it (1) can help expel most residues from any other hair products that might be found on the scalp and hair shafts; and (2) it can condition hair making it softer, silkier, and shinier. Such benefits might be attributed to APCV’s acetic acid content [24].

Nano-particulate systems are newly emerging drug delivery systems that are widely investigated, either for topical or systemic application [25,26,27,28]. Nanoemulsions are recognized as clear, meta-stable mixtures of two immiscible liquids (mainly water and oil) firmed by a surfactant/cosurfactant interfacial film [29]. Nanoemulsions display several merits over some other dosage forms as: (1) a higher absorption rate with lower variation in absorption; (2) anti-oxidant and hydrolysis protection effects; (3) delivery of lipophilic and hydrophilic drugs; (4) enhanced water solubility for poorly soluble agents; (5) boosted bioavailability; (6) minimized required doses and side effects; (7) no toxicity or irritation observed when used on skin or mucosal membranes; (8) controlled drug release; and (9) better ex vivo permeation [30]. Some of those merits are due to the globular size that locate in nanosize range, others due to the presence of surfactant within the formula, and other could be due to the specific actions related to the incorporated oil type within the prepared nanoemulsion.

Several research groups have investigated the nanoemulsions of essential oils and their beneficial outstanding results [31,32]. Statistical experimental designs were established to obtain the greatest amount of information while also employing the fewest number of experiments, identifying the interactions between variables, and explaining the reasons behind any experimental errors [33]. An extra benefit of using these designs is that they demand careful elaboration and adhering to statistical laws. This stringency forces researchers to be precise in determining the experimental objectives and methodologies needed to achieve those objectives. Moreover, the design can predict the optimum formulation behavior and how to scale it up [34]. Statistical designs are an economic way of researching, because they often provide the best solution for the formulation [35].

Based on the aforementioned information, the purpose of this study was to improve treatment for alopecia areata by (1) implementing statistical experimental designs, and (2) by applying the hair-growth-promoting and -protecting effects of GO and APCV to enhance the solubility and permeation of MX by incorporating it into a nanoemulsion utilizing GO as the oil phase and APCV as the aqueous phase.

## 2. Materials and Methods

### 2.1. Materials

Garlic oil was donated by the Verywell Health Company (New York, NY, USA). Tween 20 and Span 20 were purchased from the BASF SE Chemicals Company (Ludwigshafen, Germany). Apple cider vinegar was procured from Jiangxi Origin Aromatics Co., Ltd. (Xi’an, China). Minoxidil was gifted by Qingdao Sigma Chemical Co., Ltd. (Qingdao, China). Transcutol was donated by Gattefosse (Saint-Priest, France). Methanol, acetonitrile, and phosphate HPLC-grade buffers were purchased from Sigma Aldrich (St. Louis, MO, USA).

### 2.2. Estimation of Required Hydrophilic Lipophilic Balance (RHLB) for Garlic Oil in Apple Cider Vinegar

The reported RHLB for GO in water is 14 [36]. Different ratios (i.e., 0.57:0.43, 0.52:0.48, 0.45:0.55, 0.39:0.61, 0.33:0.67, 0.27:0.73, 0.20:0.8, 0.14:0.86, and 0.085:0.915) of Span 20 and Tween 20 respectively were used to obtain the optimal RHLB of the surfactant mixture (ranging from 12 to 16) to disperse GO (oil phase) in APCV (aqueous phase) for nanoemulsion formulation as follows:

Firstly, 0.15 g of GO were vortexed with 0.35 g of surfactant mixture (Smix) to prepare the organic phase. Next, it was added to an aqueous phase (0.5 g of APCV) dropwise and then vortexed to formulate a preliminary emulsion. The formed emulsions’ droplet size was determined using Zetatrac by Microtrac (Montgomeryville, PA, USA) [37] to assign the most suitable RHLB of surfactant mixture to develop a GO emulsion in APCV. Experiments were conducted thrice.

### 2.3. Pseudoternary Phase Diagram Construction

Determining the right levels of oil, surfactant, and co-surfactant is crucial for preparing a stable and robust nanoemulsion. The pseudoternary phase diagram tool was employed to determine the optimal nanoemulsion region for the components: GO (oil phase), surfactant mixture in the ratio of 0.39:0.61, and Transcutol as the co-surfactant, using aqueous titration procedure and the formation of nanoemulsioms was evidenced by visual inspection [31]. The total composition of the three ingredients was kept at 100% with 50 mg MX. Several combinations were allocated to find the nanoemulsion region in the diagram.

### 2.4. Preparation and Optimization of the MX-Loaded GO-APCV Nanoemulsion

A distance coordinate exchange quadratic mixture design was used to inspect the influence of varying formulation parameters on the GO-APCV nanoemulsion features, using Design-Expert^®^ software (version 13.0.7.0, Stat-Ease Inc., Minneapolis, MN, USA). Three independent factors were assessed for this purpose: GO percentage in the range of 5–18% (A); Smix (surfactant:co-surfactant, 2:1) in the range of 20–50% (B); and APCV percentage in the range of 32–75% (C) (Table 1). Each formulation contained 50 mg MX. The droplet size, drug steady state flux (MX Jss), and minimum effective concentration (MIC) of nanoemulsion against *P. acnes* were considered the dependent variables for the evaluation and optimization of the MX-loaded GO-APCV nanoemulsion formulations. A total of 18 runs were developed randomly (Table 2). About 1 g of each mixture was prepared by mixing the three components (oil, Smix, and APCV).

### 2.5. Preparation of MX-GO-APCV Nanoemulsion

Definite proportions of the three components of MX-GO-APCV nanoemulsion namely, GO (oil), Smix prepared from Tween 20/Span 20 mixed in the ratio 0.39/0.61 (surfactant mixture): Transcutol (cosurfactant) in the ratio 2:1, and APCV (aqueous phase) formulations were mixed and vortexed for 5 min. Finally, the mixtures were set aside to equilibrate for 12 h in a shaking water bath set at 100 rpm and 37 °C [30].

### 2.6. Evaluation of the MX-GO-APCV Nanoemulsion

#### 2.6.1. Determination of MX-GO-APCV Nanoemulsion Droplet Size

The average droplet size of MX-GO-APCV nanoemulsions were examined through dynamic light scattering technique (DLS) (Zetatrac, Microtrac, Montgomeryville, PA, USA). Initially, nanoemulsion samples were diluted with distilled water (1:10) to revoke the multi-scattering effect, reduce the expected formulation viscosity and to provide precise comparison between different formulations [37]. The particle size data was reported as the Z-average mean diameter and the Zaverage particle diameter was obtained based on distribution by number which was gathered by the software. All measurements were taken in triplicate at ambient temperature (25 °C ± 2 °C) and an angle of 90° to the incident beam [38].

#### 2.6.2. Ex Vivo Skin Permeation Study of MX-GO-APCV Nanoemulsion

Ex vivo permeation studies were accomplished employing Microette Plus Hanson Automated Vertical Diffusion Cells (Hanson Research, Chatsworth, CA, USA) according to a previously described procedure [26]. The receptor compartment contained phosphate buffer saline (PBS, pH 5.8) as the receptor milieu and was maintained at 32 °C ± 0.5 °C with a stirring rate of 400 rpm. The permeation membrane was obtained from male Wistar rats whose abdominal regions were shaved by electric clippers, and whose skin was removed (3 × 3 cm^2^) and cleared from subcutaneous fats. Donor chamber contained 4 mL of emulsion equivalent to 14 mg of MX and the receiver chamber contained 10 mL of receptor media, while the surface area for permeation was 2.5 cm^2^. The animals were provided by the Cairo Agriculture Center for Experimental Animals, Cairo, Egypt. Ethical approval for this study was obtained from The Research Ethics Committee of the ACM Center for Clinical Laboratories, Cairo, Egypt, following the Helsinki agreement protocol and the Guiding Principle in Care and Use of Animals (DHEW publication NIH 80-23), approval No. (293-07-21) at July 2021. A magnifier was used to examine the integrity of the excised skin. Next, the skin was soaked in PBS (pH 5.8) for 3 h, after which the prepared skin was inserted between the donor and receptor compartments of the cells. Permeation of MX-GO-APCV nanoemulsions was assessed, and MX amounts permeated across the skin were evaluated using RP-HPLC, according to previously published, validated methods on an ODS C18 column (25 cm × 4.6 mm, 5 µ particle size) using a mobile phase composed of methanol:water (70:30 *v*/*v*) in addition to 0.5 % triethylamine (TEA). The pH was adjusted to 6.38 with orthophosphoric acid (OPA). The flow rate was 1 mL/min, and eluents were detected by a UV detector at 210 nm. The percentage of MX permeated at each sampling time was determined, and the steady state flux (Jss) for each sample was obtained as follows [39]:

Jss = dQ/dt(1)

where Q (µg/cm^2^) is the cumulative amount permeated through the unit area of the membrane surface. Measurements were made in triplicate.

#### 2.6.3. Assessment of MIC of the Prepared MX-GO-APCV Nanoemulsion against *P. acnes*

*P. acnes*, was obtained from the Agricultural Research Service (NRRL, Peoria, IL, USA). Prior to the assay, the bacterium was incubated in Mueller Hinton broth (Mast Group Ltd., Merseyside, UK) at 37 °C for 72 h under anaerobic conditions [40]. A 96-well microplate assay was used to determine the antibacterial activity of nanoemulsion samples against *P. acnes* according to a previously described method [41] with minor modifications. The bacterium culture obtained after 72 h was dispersed in Mueller Hinton broth, and the dispersion was curbed to 0.5 McFarland turbidity standard. All samples were then dissolved in 5% dimethyl sulfoxide (DMSO) and subsequently diluted with the used broth, up to a concentration of 100 μg/mL. Further, 100 μL of each sample was poured into the 96-well plate. The same procedure was adopted with the positive control (100 μg/mL erythromycin) and with the negative control (5% DMSO). Next, 100 μL of bacterial suspension was added to each well. The minimum inhibitory concentrations (i.e., the lowest concentration of the sample at which the microorganism demonstrates no visible growth) for the tested samples and the positive control were determined. Twofold serial dilutions were performed by adding culture broth to obtain concentrations within the range of 0.5 to 50 μg/mL of the tested sample, while (for the positive control), dilutions from 0.003 to 100 μg/mL were used during the MIC determination. The test was performed thrice.

### 2.7. Optimization of the MX-GO-APCV Nanoemulsion

Design-Expert^®^ software (Stat-Ease, Inc., 2021 E. Hennepin Avenue, Ste 480, Minneapolis, MN 55413-2726, USA) was adopted to select the optimized nanoemulsion formulation by means of the desirability function. The purpose of the optimization process was to select a formulation with the minimum droplet size and MIC and the maximum MX Jss. The solution with the desirability value closest to 1 was chosen. To ensure model validity, the selected nanoemulsion formulation was developed, characterized, and ultimately compared to the responses valued expected by the software.

### 2.8. Preparation and Evaluation of the Optimized MX-GO-APCV Nanoemulsion

The optimized MX-GO-APCV nanoemulsion composed of 0.180% GO, 0.500% Smix, and 0.320% of APCV was prepared and evaluated for globule size, Jss, and MIC as previously described. In addition, the optimized MX-GO-APCV nanoemulsion was evaluated for stability, permeation parameters, and kinetic modeling as follows.

### 2.9. Stability Index Determination

Heat-cool stability testing was first performed to the formulation; it was kept for 48 h at 4 °C, followed by 48 h at 40 °C temperature. The cycle was repeated thrice, and then formulation was tested visually for any signs of instability.

Secondly, a freeze–thaw accelerated stability study was performed using varying temperature values to prove the thermodynamic stability of the optimized MX-GO-APCV nanoemulsion. Through the course of the study, the droplet size was determined, and then the samples were subjected to three consecutive freeze–thaw cycles (freezing at −25 °C for approximately 24 h and thawing at +25 °C for another 24 h). The droplet size was measured after each consecutive cycle. The stability index was assigned adopting the following equation [31]:

Stability index = ([Initial size − Change in size]/Initial size) × 100(2)

#### Observation on Phase Separation

Each formulation was centrifuged at 5000 rpm for 30 min at room temperature (25 °C) and observed for phase separation.

### 2.10. Analysis of Permeation Parameters and Kinetic Modeling

The release pattern of MX was determined according to the relation of the cumulative amount of MX permeated (Q) per unit area as a function of time. Steady-state flux (Jss), permeability coefficient (Pc), and diffusion coefficient (D) were calculated as previously reported. In addition, the obtained permeation data were fitted into different kinetic models: zero, first, Higuchi, Hixson–Crowell, and Baker–Lonsdale models. Linearity and correlation coefficient (r) were evaluated.

## 3. Results and Discussion

### 3.1. Estimation of Required Hydrophilic Lipophilic Balance (RHLB) for GO in APCV

As seen in Table 2, the smallest globule size of GO-APCV emulsion (210 ± 20 nm) was obtained by using a Tween 20/Span 20 mixture in the ratio 0.39:0.61. Therefore, the RHLB for GO in APCV is 13.5, because it is the RHLB corresponding to the lowest droplet size. Consequently, the Tween 20/Span 20 mixture in the ratio 0.39:0.61 was used for further studies in formulating the GO-APCV emulsion.

**Figure 1:**
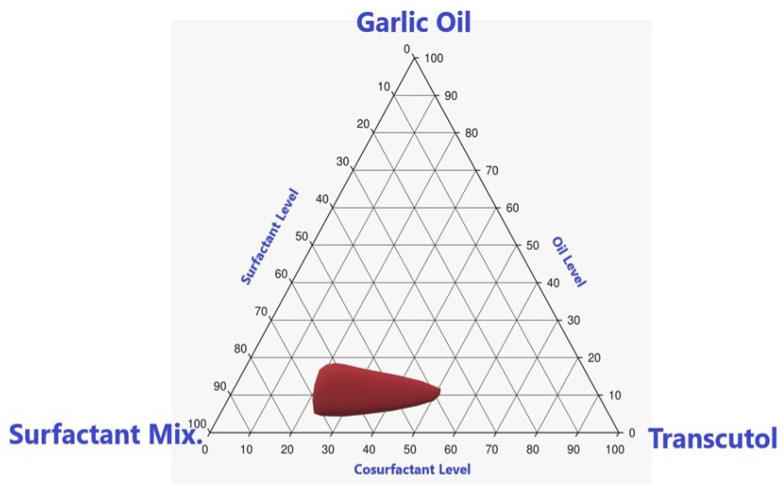
The pseudoternary phase diagram of GO, Smix, and Transcutol co-surfactant.

Notably, the nanoemulsion region indicated that GO may be used in the range of 8–18%, while the level of surfactant mixture ranged from 40% to 70% and the level of Transcutol ranged from 20% to 40% as observed in Figure 1. Therefore, a mixture of surfactant and cosurfactant (Smix) will be used in formulating the nanoemulsion by a ratio 2:1 surfactant to cosurfactant (Smix ratio will be 2:1).

### 3.2. Evaluation of the MX-GO-APCV Nanoemulsion

#### 3.2.1. Determination of MX-GO-APCV Nanoemulsion Droplet Size

Droplet size of the investigated nanoemulsions oscillated between 104 and 300 nm, as summarized in Table 3, with PDI values from 0.09 to 0.31. These values reveal the favorable stability, homogeneity, and size distribution of the prepared MX-GO-APCV nanoemulsions.

A quadratic model of polynomial analysis revealed the highest significant mean squared value exceeding the residual error (*p* < 0.0084), so it was adopted for droplet size data analysis. The investigated statistical design disclosed the adopted model’s capacity to assess the significant effect of garlic % (A), Smix % (B), and APCV % (C) on MX-GO-APCV nanoemulsions’ droplet size. The specified model acquired an adjusted R^2^ value of 0.7921, which was in line with an expected R^2^ of 0.7184. ANOVA analysis of the obtained data revealed the following equation:

Droplet size = −2.316 × 10^06^A − 11,310.02B + 146.50C + 4.475 × 10^06^AB + 4.374 × 10^06^AC + 22,973.84BC + 3.350 × 10^06^AB(A − B) + 3.078 × 10^06^AC(A − C) + 22,757.47BC(B − C) − 1.110 × 10^07^A^2^BC − 2.963 × 10^06^AB^2^C − 3.067 × 10^06^ABC^2^ + 1.323 × 10^06^AB(A − B)^2^ + 1.024 × 10^06^AC(A − C)^2^ + 14,427.31BC(B − C)^2^ (3)

**Figure 2:**
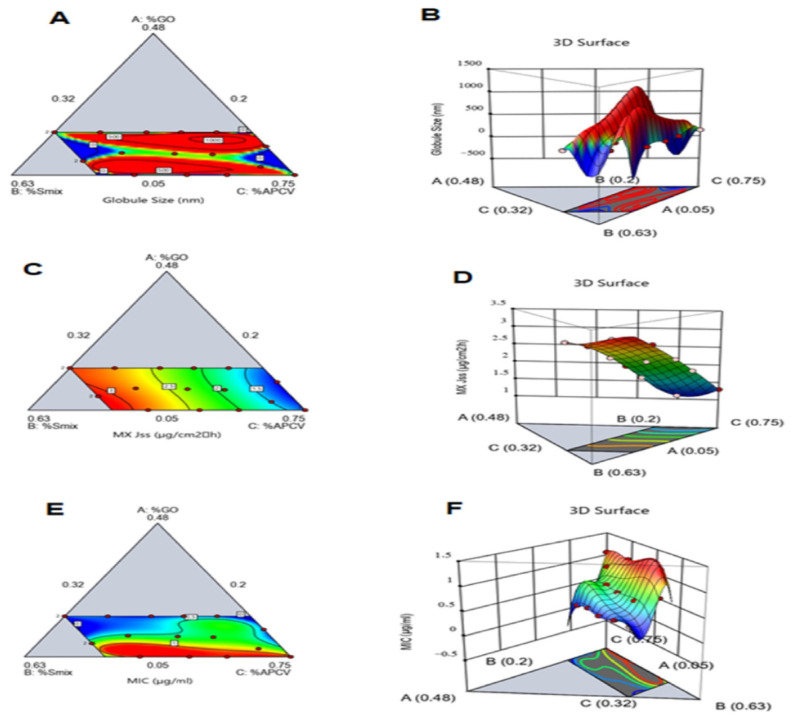
Statistical design plots for the droplet size, MX Jss, and MIC of MX-GO-APCV nanoemulsions: (**A**) contour plot for droplet size, (**B**) response surface plot for droplet size, (**C**) contour plot for MX Jss, (**D**) response surface plot for MX Jss, (**E**) contour plot for MIC, and (**F**) response surface plot for MIC.

Figure 2 shows the contour and 3D-surface plots that revealed the effect of the factors on MX-GO-APCV nanoemulsions’ droplet size, which elucidated that the nanoemulsions’ droplet size was at its lowest levels when the three factors were in their median levels. This is an expected result since the factors are inversely proportional to each other.

#### 3.2.2. Ex Vivo Skin Permeation Study of MX-GO-APCV Nanoemulsion

Steady state flux of MX (MX Jss) of the studied nanoemulsions through skin exhibited values between 1.3 and 3.1 µg/cm^2^ h, as summarized in Table 3.

A special quartic model of polynomial analysis articulated the highest significant mean squared value exceeding the residual error (*p* < 0.0002), so it was adopted for MX Jss data analysis. The explored experimental design revealed the statistical model’s capacity to evaluate the significant effect of garlic % (A), Smix % (B), and APCV % (C) on MX Jss of MX-GO-APCV nanoemulsions. The allocated model gained an adjusted R^2^ value of 0.9992, which was in close agreement with an expected R^2^ of 0.9984. ANOVA analysis of the obtained data revealed the following equation:

(4) $$\mathrm{MX}\;\mathrm{Jss}=+8.31A+3.76B+1.30C-10.14\mathrm{AB}-10.01\mathrm{AC}+0.7290\mathrm{BC}-29.18A^{2}\mathrm{BC}+20.19{\mathrm{AB}}^{2}C+14.32{\mathrm{ABC}}^{2}$$

Figure 2 shows the contour and 3D-surface plots that revealed the factors’ effect on MX-GO-APCV nanoemulsions MX Jss, which clarifies that the Smix % (B) was the factor with the main effect on MX Jss. The figures demonstrate that increasing Smix % resulted in a higher MX Jss. Such a result can be understood in the light of the Smix effect on MX-GO-APCV nanoemulsions’ droplet size, as it is expected that the higher the Smix %, the lower the droplet size of nanoemulsions. Therefore, a larger surface area will be offered for higher flux to take place. Moreover, the surfactants and co-surfactant comprising the Smix are thought to have a skin permeation-enhancing effect. Non-ionic surfactants usually play their role as penetration enhancers through different mechanisms. Of these, the ability to permeate through the intercellular space in stratum corneum, enhancing its fluidity and solubilizing and extracting lipid components. Furthermore, surfactants can penetrate the intercellular matrix and interact and engage with keratin filaments leading to corneocyte disruption. Several research groups reported similar findings [42,43].

#### 3.2.3. Assessment of MIC of the Prepared MX-GO-APCV Nanoemulsion against *P. acnes*

MIC of the developed nanoemulsion against *P. acnes* fluctuated between 0.203 and 1.124 µg/mL, as summarized in Table 3.

The quadratic model of polynomial analysis was the statistical model that showed the most significant mean squared value exceeding the residual error (*p* < 0.0001). Therefore, this model was used to analyze MIC data. The applied experimental design explained the independent variables’ effect on MIC values of MX-GO-APCV nanoemulsions. The determined model had a predicted R^2^ value of 0.9889, which was in close accordance with adjusted R^2^ of 0.9931. ANOVA analysis of obtained data revealed the following equation:

(5) $$\begin{array}{ll} \mathrm{MIC}= & -762.16A-7.84B+1.07C+1501.49\mathrm{AB}+1426.38\mathrm{AC}+18.17\mathrm{BC}+1173.76\mathrm{AB}\left(A-B\right)\\ & +981.86\mathrm{AC}\left(A-C\right)+17.99\mathrm{BC}\left(B-C\right)-3763.51A^{2}\mathrm{BC}-974.63{\mathrm{AB}}^{2}C-1032.63{\mathrm{ABC}}^{2}\\ & +526.15\mathrm{AB}{\left(A-B\right)}^{2}+311.22\mathrm{AC}{\left(A-C\right)}^{2}+10.93\mathrm{BC}{\left(B-C\right)}^{2} \end{array}$$

Figure 2 shows the contour and 3D-surface plots that revealed the effect of the factors on MX-GO-APCV nanoemulsions’ MIC. It was observed that GO % and Smix % exhibited the most prominent effects on MIC response. These findings could be justified based on the composition of GO and the unique properties of Smix components. The outstanding antimicrobial effect of GO was attributed to its different sulfides content, which is capable of destroying the bacterial cell wall by interfering with the sulfhydryl groups of bacterial cells’ proteins to yield mixed disulfides. Previous research outcomes supported these findings [44]. Additionally, the allyl group(s) that is abundant in GO, plays a substantial role in recognizing the antibacterial action of GO [45]. The effect of Smix in decreasing MIC values could be attributed to its membrane-fluidizing activity. It is possible that the non-ionic surfactants could interfere with the phospholipid hydrocarbon present in bacterial cell walls, therefore leading to increased membrane fluidity and eventually cell lysis.

### 3.3. Optimization of MX-GO-APCV Nanoemulsion Formulations

**Figure 3:**
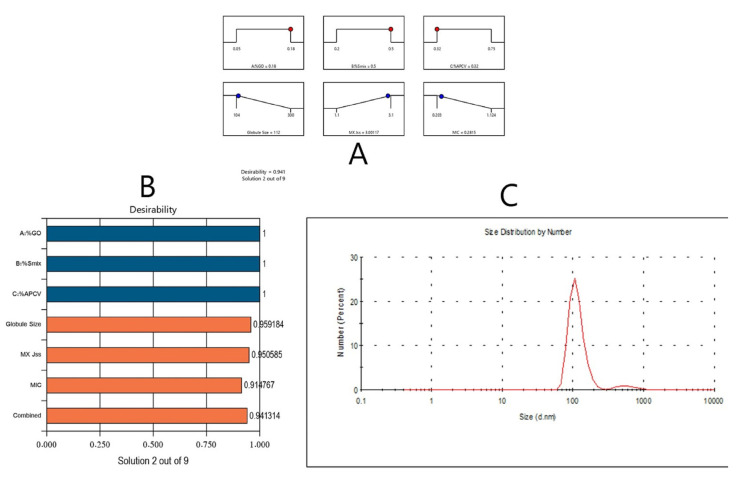
Bar chart, desirability ramp, and DLS distribution for the optimization process. The desirability ramp illustrates the levels of study factors and expected values for the dependent variables of the optimized OEO-SNED (**A**). The bar chart illustrates the values of desirability for the conjugated responses (**B**). The DLS distribution indicated droplet size for the optimized formula (**C**).

Optimizing an experimental procedure usually involves identify the independent variables’ levels that are most suitable for producing pharmaceutical products with the optimal desired responses [19]. Therefore, the global desirability function (D) was applied to optimize the data obtained from the statistical design generated by Design-Expert^®^ software. The studied responses were set to certain limits (droplet size, MIC to minimum, and MX Jss to a maximum) to construct an overlay graph and optimize the studied factors. A desirability value of 0.941 for responses in the desirability plot was obtained. The optimum formulation consisted of 0.180% of GO, 0.500% of Smix, and 0.320% of APCV. The manufactured optimized formulation had a droplet size of 110 nm, MX Jss of 3 μg/cm^2^·h and MIC of 0.275 μg/mL. Figure 3 illustrates the desirability ramp that clarifies the optimal levels for investigated factors and expected values of the dependent variables of the optimum formulation. No major differences were observed upon comparing the experimental and predicted values of the optimized formulation’s responses (*p* > 0.05), which affirms the precision and validity of the generated equations (see Table 4).

Interestingly, Factors A and B (i.e., GO % and Smix %, respectively) were set at their highest levels in optimum formulation, while factor C (APCV %) was set at its lowest level. This result is favorable because it is advantageous to have GO % in its maximum concentration due to GO’s various beneficial effects in decreasing the MIC required for resisting the bacterial infections, enhancing MX permeation, and nourishing hair shafts. Similar interpretations were found in other literature [46]. Moreover, it is desirable to have Smix in its highest level because it exerts a prominent effect on nanoemulsion droplet size, causing it to decrease to the required nano-level. Such reduction in droplet size allows for greater permeation of the drug to occur, because it offers a larger surface area for the diffusion process through the skin [47]. Additionally, higher ratios of Smix provide greater protection against microbial infection, as was previously described and interpreted by other researchers [48].

### 3.4. Evaluation of the Optimized Formulation

#### 3.4.1. Determination of Stability Index

No change in formulation consistency was observed following the heat–cool test. The stability index is a parameter of utmost importance in exploring the stability of nanoemulsions. The high value of stability index obtained (96%) for optimized formulation indicates that the developed optimal nanoemulsion acquired reasonable stability and that the optimal levels specified by the design were able to form a quality, stable nanoemulsion [31]. No phase separation was observed upon centrifugation of optimized sample.

#### 3.4.2. Analysis of Permeation Parameters of Optimum Formulation

The ex vivo permeation results indicate that incorporating MX into nanoemulsion drug delivery systems enhances the drug’s permeation by at least 2.12-fold compared to the drug in aqueous dispersion, such an outcome clarifies that the nanoemulsions play a substantial role in amplifying MX permeation through skin. GO (which was used within the nanoemulsion formulation as an oil phase) enhanced the MX permeation and steady state flux in contrast to the optimum formulation, which contained no GO. This result could be due to the permeability-enhancing action of the oil brought about by changing and manipulating stratum corneum features, either by increasing MX solubility and partitioning through skin or by enhancing membranes’ fluidity, as has previously been reported in literature [49].

Moreover, the presence of APCV within the nanoemulsion as an aqueous phase enhanced the MX permeation and steady state flux compared to the optimum formulation prepared without APCV. This result presented in Table 5, indicates that APCV has penetrating-enhancing abilities, but less than that of GO. Collectively, the presence of GO and APCV within the optimized nanoemulsion formulation has a synergistic-enhancing activity on the MX permeation across the skin membrane, and the percent permeated increased from 12.7% to 41.6%.

#### 3.4.3. Kinetic Analysis of the Permeation Data

The model that best fits the permeation data is selected based upon the correlation coefficient (R) value in various models. The model that gives a high R value is considered to be the best fit of the permeation data. It was observed that optimized nanoemulsions followed Hixson–Crowell release kinetics (0.9802), which this model explains, whether the permeation pattern follows Fickian diffusion or not (which can only be judged on the basis of the value of n where n is estimated from linear regression). It was observed that the MX-GO-APCV nanoemulsion followed the Korsmeyer–Peppas model, and the value of release exponent (n) obtained for the formulations was found to be n = 1.0124, which is higher than 0.89. This result implies that the permeation of MX from the system follows super case II transport.

## 4. Conclusions

MX was successfully incorporated into a nanoemulsion with satisfying properties. A pseudoternary phase diagram was adopted to assign the concentrations of GO, surfactants, and co-surfactant mixtures to determine the most convenient nanoemulsion regions to develop the required drug delivery system. The droplet size of the manufactured nanoemulsions oscillated between 104 and 280 nm with proper homogeneity. MX Jss and MIC of developed formulations fluctuated between 3 and 3.1 µg/cm^2^ h and from 0.203 to 1.124 µg/mL, respectively. Additionally, optimized formulation acquired a reasonable stability (96%). Furthermore, the optimized MX-GO-APCV nanoemulsion showed enhanced ex vivo permeation parameters compared to varying formulations. Finally, the optimum formulation followed the Korsmeyer–Peppas model in the permeation study, implying that the permeation of MX from the system follows super case II transport. Thus, this study clarifies that an MX-GO-APCV nanoemulsion could be an effective nanoplatform that would provide good permeation of MX and maximize its beneficial effects in treating alopecia areata.

## Figures and Tables

**Table 1:** Experimental plan for MX-loaded GO-APCV nanoemulsion.

Independent Variables	Dependent Variables		Constraints	
	−1	+1		
GO % (A)	5	18	Droplet Size(Y_1_) (nm)	Minimum
Smix % (B)	20	50	MX Jss (Y_2_) (μg/cm^2^ h)	Maximum
APCV % (C)	32	75	MIC (Y_3_) (μg/mL)	Minimum

**Table 2:** RHLB and droplet sizes of GO-APCV emulsions formulated using different Tween 20/Span 20 ratios.

RHLB	Tween 20 Ratio	Span 20 Ratio	Droplet Size of Formed GO-APCV Emulsion
12	0.57	0.43	550 ± 40 nm
12.5	0.52	0.48	435 ± 34 nm
13	0.45	0.55	280 ± 28 nm
13.5	0.39	0.61	210 ± 20 nm
14	0.33	0.67	295 ± 27 nm
14.5	0.27	0.73	360 ± 36 nm
15	0.20	0.80	410 ± 33 nm
15.5	0.14	0.86	470 ± 41 nm
16	0.085	0.915	580 ± 49 nm

**Table 3:** Distance coordinate exchange quadratic mixture design responses of MX-GO-APCV nanoemulsions.

Component 1		Component 2	Component 3	Y_1_	Y_2_	Y_3_	
Run	A: %GO	B: %Smix	C: %APCV	Globule Size	MX Jss	MIC	PDI
				(nm)	(μg/cm^2^ h)	(μg/mL)	
1	0.0756	0.2392	0.685	270	1.5	0.854	0.12
2	0.05	0.4436	0.5063	104	2.9	1.124	0.17
3	0.18	0.3603	0.4596	120	2.4	0.203	0.23
4	0.18	0.4305	0.3894	154	2.8	0.266	0.3
5	0.18	0.3078	0.5121	253	2.1	0.261	0.19
6	0.1369	0.2	0.663	300	1.1	0.318	0.22
7	0.05	0.2	0.75	147	1.3	1.075	0.25
8	0.05	0.3637	0.5862	115	2.4	1.101	0.18
9	0.1173	0.4291	0.4535	169	2.8	0.552	0.09
10	0.18	0.5	0.32	114	3	0.282	0.31
11	0.0925	0.5	0.4074	105	3.1	0.702	0.3
12	0.18	0.5	0.32	110	3	0.281	0.26
13	0.05	0.3028	0.6471	141	2	1.083	0.11
14	0.0925	0.5	0.4074	106	3.1	0.702	0.13
15	0.18	0.2537	0.5662	280	1.7	0.206	0.29
16	0.05	0.2	0.75	146	1.3	1.074	0.21
17	0.115	0.35	0.535	216	2.3	0.563	0.25
18	0.1127	0.2936	0.5936	253	1.9	0.622	0.3

**Table 4:** Actual and experimental values of the optimized nanoemulsion formulation.

Solution	GO %	Smix %	APCV %	Droplet Size (nm)	MX Jss (μg/cm^2^ h)	MIC (μg/mL)	Desirability
Predicated value	0.180	0.500	0.320	112	3.001	0.281	0.941
Experimental value	0.180	0.500	0.320	110	3	0.275	0.941

**Table 5:** Ex vivo permeation results of optimized formulation.

Permeation Parameters	Optimized Formulation	Optimized Formulation Prepared with Oleic Acid Instead of GO	Optimized Formulation Prepared with Distilled Water Instead of APCV	MX Aqueous Suspension
Cumulative amount permeated, Q, (μg/cm^2^) ^a^	2315 ± 243	1511 ± 112	1894 ± 192	711 ± 87
Cumulative percentage permeated	41.6%	27.2%	34.1%	12.7%
Steady state flux, Jss, (μg/cm^2^·h) ^b^	3 ± 0.15	2.1 ± 0.11	2.4 ± 0.21	1.11 ± 0.12
Permeability coefficient, P, (cm/h) ^c^	2.142 × 10^−4^	1.5 × 10^−4^	1.714 × 10^−4^	0.785 × 10^−4^
Diffusion coefficient, D, (cm^2^/h) ^d^	8.11 × 10^−5^	5.93 × 10^−5^	6.71 × 10^−5^	5.66 × 10^−5^
Enhancement factor (EF) ^e^	3.25	2.12	2.66	-

N.B. ^a^: Q = the cumulative amount permeated through the unit area of the membrane surface; ^b^: Jss = Calculated from slop of curve plotted between Q and time; ^c^: P = Mx Jss/original concentration; ^d^: D = Calculated from the slop of curve plotted between Q and square root of time; ^e^: EF: Q of studied formulations/Q of drug suspension.

## Data Availability

All data available are reported in the article.

## References

[1] Epstein E. (2001). Evidence-based treatment of alopecia areata. J. Am. Acad. Dermatol..

[2] Sharma V.K., Dawn G., Kumar B. (1996). Profile of alopecia areata in northern India. Int. J. Dermatol..

[3] Strober B.E., Siu K., Alexis A.F., Kim G., Washenik K., Sinha A., Shupack J.L. (2005). Etanercept does not effectively treat moderate to severe alopecia areata: An open-label study. J. Am. Acad. Dermatol..

[4] Xing L., Dai Z., Jabbari A., Cerise J.E., Higgins C.A., Gong W., de Jong A., Harel S., DeStefano G.M., Dai L.R. (2014). Alopecia areata is driven by cytotoxic T lymphocytes and is reversed by JAK inhibition. Nat. Med..

[5] McElwee K.J., Boggess D., Olivry T., Oliver R.F., Whiting D., Tobin D.J., Bystryn J.-C., King L.E., Sundberg J.P. (1998). Comparison of alopecia areata in human and nonhuman mammalian species. Pathobiology.

[6] Wang E., Lee J.S., Hee T.H. (2012). Is propionibacterium acnes associated with hair casts and alopecia?. Int. J. Trichology.

[7] Iwata C., Akimoto N., Sato T. (2005). Augmentation of lipogenesis by 15 deoxy-Ä12,14—prostaglandin J2 in hamster sebaceous glands: Identification of cytochrome P450-mediated by 15 deoxy-Ä12,14 -prostaglandin J2 production. J. Investig. Dermatol..

[8] Brüggerman H. (2005). Insights in the pathogenic potential of Propionibacterium acnes from its complete genome. J. Cutan. Med. Surg..

[9] Young J.W., Conte E.T., Leavitt M.L., Nafz M.A., Scroeter A.L. (1991). Cutaneous immunopathology of androgenetic alopecia. J. Am. Osteopath Assoc..

[10] Suchonwanit P., Thammarucha S., Leerunyakul K. (2019). Minoxidil and its use in hair disorders: A review. Drug Des. Dev. Ther..

[11] Mura S., Manconi M., Sinico C., Valenti D., Fadda A.M. (2009). Penetration enhancer-containing vesicles (PEVs) as carriers for cutaneous delivery of monoxidil. Int. J. Pharm..

[12] Aronson J.K., Aronson J.K. (2006). Minoxidil. Meyler’s Side Effects of Drugs: The International Encyclopedia of Adverse Drug Reactions and Interactions.

[13] Pavithran K. (1993). Erythema multiforme following topical minoxidil. Indian J. Dermatol. Venerol. Leprol..

[14] Wagner L., Kenreigh C., Enna S.J., David B.B. (2007). Minoxidil. xPharm: The Comprehensive Pharmacology Reference.

[15] Padois K., Cantiéni C., Bertholle V., Bardel C., Pirot F., Falson F. (2011). Solid lipid nanoparticles suspension versus commercial solutions for dermal delivery of minoxidil. Int. J. Pharm..

[16] Bayan L., Koulivand P.H., Gorji A. (2014). Garlic: A review of potential therapeutic effects. Avicenna J. Phytomed..

[17] Satyal P., Craft J.D., Dosoky N.S., Setzer W.N. (2017). The Chemical Compositions of the Volatile Oils of Garlic (Allium sativum) and Wild Garlic (Allium vineale). Foods.

[18] El-Sayed H.S., Chizzola R., Ramadan A.A., Edris A.E. (2017). Chemical composition and antimicrobial activity of garlic essential oils evaluated in organic solvent, emulsifying, and self-microemulsifying water based delivery systems. Food Chem..

[19] Ankri S., Mirelman D. (1999). Antimicrobial properties of allicin from garlic. Microbes Infect..

[20] Rnault I., Haffner T., Siess M.H., Vollmar A., Kahane R., Auger J. (2005). Analytical method for appreciation of garlic therapeutic potential and for validation of a new formulation. J. Pharm. Biomed Anal..

[21] Piyasena P., Rayner M., Bartlett F.M., Lu X., McKellar R.C. (2002). Characterization of Apples and Apple Cider Produced by a Guelph Area Orchard. LWT—Food Sci. Technol..

[22] Renard C.M.G.C., Le Quéré J.M., Bauduin R., Symoneaux R., Le Bourvellec C., Baron A. (2010). Modulating polyphenolic composition and organoleptic properties of apple juices by manipulating the pressing conditions. Food Chem..

[23] Yagnik D., Serafin V.J., Shah A. (2018). Antimicrobial activity of apple cider vinegar against Escherichia coli, Staphylococcus aureus and Candida albicans; downregulating cytokine and microbial protein expression. Sci. Rep..

[24] Tang S.C., Yang J.H. (2018). Dual Effects of Alpha-Hydroxy Acids on the Skin. Molecules.

[25] Salem H.F., Nafady M.M., Ewees M.G.E., Hassan H., Khallaf R.A. (2021). Rosuvastatin calcium-based novel nanocubic vesicles capped with silver nanoparticles-loaded hydrogel for wound healing management: Optimization employing Box-Behnken design: In vitro and in vivo assessment. J. Liposome Res..

[26] Salem H.F., El-Menshawe S.F., Khallaf R.A., Rabea Y.K. (2020). A novel transdermal nanoethosomal gel of lercanidipine HCl for treatment of hypertension: Optimization using Box-Benkhen design, in vitro and in vivo characterization. Drug Deliv. Transl. Res..

[27] Khallaf R.A., Aboud H.M., Sayed O.M. (2020). Surface modified niosomes of olanzapine for brain targeting via nasal route; preparation, optimization, and in vivo evaluation. J. Liposome Res..

[28] Ali S.A., Sindi A.M., Maira Y.H., Khallaf R.A. (2021). Oral gel loaded by ethotransfersomes of antifungal drug for oral thrush: Preparation, characterization, and assessment of antifungal activity. J. Drug Del. Sci. Technol..

[29] Hosny K.M., Alhakamy N.A., Sindi A.M., Khallaf R.A. (2020). Coconut Oil Nanoemulsion Loaded with a Statin Hypolipidemic Drug for Management of Burns: Formulation and In Vivo Evaluation. Pharmaceutics.

[30] Hosny K.M., Sindi A.M., Alkhalidi H.M., Kurakula M., Hassan A.H., Bakhaidar R.B., Abualsunun W.A., Almehmady A.M., Khames A., Rizg W.Y. (2021). Development of omega-3 loxoprofen-loaded nanoemulsion to limit the side effect associated with NSAIDs in treatment of tooth pain. Drug Deliv..

[31] Hosny K.M., Khallaf R.A., Asfour H.Z., Rizg W.Y., Alhakamy N.A., Sindi A.M., Alkhalidi H.M., Abualsunun W.A., Bakhaidar R.B., Almehmady A.M. (2021). Development and Optimization of Cinnamon Oil Nanoemulgel for Enhancement of Solubility and Evaluation of Antibacterial, Antifungal and Analgesic Effects against Oral Microbiota. Pharmaceutics.

[32] Hosny K., Asfour H., Rizg W., Alhakamy N.A., Sindi A., Alkhalidi H., Abualsunun W., Bakhaidar R., Almehmady A.M., Akeel S. (2021). Formulation, Optimization, and Evaluation of Oregano Oil Nanoemulsions for the Treatment of Infections Due to Oral Microbiota. Int. J. Nanomed..

[33] Gregg Stetsko Manager (1986). Statistical Experimental Design and its Application to Pharmaceutical Development Problems. Drug Dev. Indust Pharm..

[34] Hosny K.M., Rizg W.Y., Khallaf R.A. (2020). Preparation and Optimization of In Situ Gel Loaded with Rosuvastatin-Ellagic Acid Nanotransfersomes to Enhance the Anti-Proliferative Activity. Pharmaceutics.

[35] Alkhalidia H.M., Naguib G.H., Kurakula M., Hamed M.T., Attar M.H., Almatrook Z.H., Aldryhim A.Y., Bahmdan R.H., Khallaf R.A., el Sisi A.M. (2018). In vitro and preclinical assessment of factorial design based nanoethosomal transdermal film formulation of mefenamic acid to overcome barriers to its use in relieving pain and inflammation. J. Drug Del. Sci. Technol..

[36] Long Y., Huang W., Wang Q., Yang G. (2020). Green synthesis of garlic oil nanoemulsion using ultrasonication technique and its mechanism of antifungal action against Penicillium italicum. Ultrason. Sonochem..

[37] Purkait A., Worede R.E., Baral D., Hazra D.K. (2020). Development of nanoemulsion formulation of mustard oil, its chemical characterization and evaluation against post harvest anthracnose pathogens. Indian Phytopathol..

[38] Abou-Taleb H.A., Khallaf R.A., Abdel-Aleem J.A. (2018). Intranasal niosomes of nefopam with improved bioavailability: Preparation, optimization, and In-Vivo evaluation. Drug Des. Dev. Ther..

[39] Zillich O.V., Schweiggert-Weisz U., Hasenkopf K., Eisner P., Kerscher M. (2013). Release and in vitro skin permeation of polyphenols from cosmetic emulsions. Int. J. Cosmet. Sci..

[40] Kumar R., Mishra A.K., Dubey N.K., Tripathi Y.B. (2007). Evaluation of Chenopodium ambrosioides oil as a potential source of antifungal, antiaflatoxigenic and antioxidant activity. Int. J. Food Microbiol..

[41] Mapunya M.B., Hussein A.A., Rodriguez B., Lall N. (2011). Tyrosinase activity of Greyia flanaganii (Bolus) constituents. Phytomedicine.

[42] Som I., Bhatia K., Yasir M. (2012). Status of surfactants as penetration enhancers in transdermal drug delivery. J. Pharm. Bioallied Sci..

[43] Shin S.C., Cho C.W., Oh I.J. (2001). Effects of non-ionic surfactants as permeation enhancers towards piroxicam from the poloxamer gel through rat skins. Int. J. Pharm..

[44] Lu X., Rasco B.A., Jabal J.M., Aston D.E., Lin M., Konkel M.E. (2011). Investigating antibacterial effects of garlic (Allium sativum) concentrate and garlic-derived organosulfur compounds on Campylobacter jejuni by using Fourier transform infrared spectroscopy, Raman spectroscopy, and electron microscopy. Appl. Environ. Microbiol..

[45] Casella S., Leonardi M., Melai B., Fratini F., Pistelli L. (2013). The role of diallyl sulfides and dipropyl sulfides in the In Vitro antimicrobial activity of the essential oil of garlic, *Allium sativum* L., and leek, *Allium porrum* L.. Phytother. Res..

[46] Hajheydari Z., Jamshidi M., Akbari J., Mohammadpour R. (2007). Combination of topical garlic gel and betamethasone valerate cream in the treatment of localized alopecia areata: A double-blind randomized controlled study. Indian J. Dermatol. Venereol. Leprol..

[47] Abdelbary A., Salem H.F., Khallaf R.A., Ali A.M. (2017). Mucoadhesive niosomal in situ gel for ocular tissue targeting: In Vitro and In Vivo evaluation of lomefloxacin hydrochloride. Pharm. Dev. Technol..

[48] Glover R.E., Smith R.R., Jones M.V., Simon K., Jackson S.K., Rowlands C.C. (1999). An EPR investigation of surfactant action on bacterial membranes. FEMS Microbiol. Lett..

[49] Jiang Q., Wu Y., Zhang H., Liu P., Yao J., Yao P., Chen J., Duan J. (2017). Development of essential oils as skin permeation enhancers: Penetration enhancement effect and mechanism of action. Pharm. Biol..

